# Decomposition in an extreme cold environment and associated microbiome—prediction model implications for the postmortem interval estimation

**DOI:** 10.3389/fmicb.2024.1392716

**Published:** 2024-05-13

**Authors:** Lavinia Iancu, Andrea Bonicelli, Noemi Procopio

**Affiliations:** ^1^Department of Criminal Justice, University of North Dakota, Grand Forks, ND, United States; ^2^Research Centre for Field Archaeology and Forensic Taphonomy, School of Law and Policing, Preston, United Kingdom

**Keywords:** postmortem interval, microbiome, prediction model, extreme environment, North Dakota

## Abstract

**Introduction:**

The accurate estimation of postmortem interval (PMI), the time between death and discovery of the body, is crucial in forensic science investigations as it impacts legal outcomes. PMI estimation in extremely cold environments becomes susceptible to errors and misinterpretations, especially with prolonged PMIs. This study addresses the lack of data on decomposition in extreme cold by providing the first overview of decomposition in such settings. Moreover, it proposes the first postmortem microbiome prediction model for PMI estimation in cold environments, applicable even when the visual decomposition is halted.

**Methods:**

The experiment was conducted on animal models in the second-coldest region in the United States, Grand Forks, North Dakota, and covered 23 weeks, including the winter months with temperatures as low as −39°C. Random Forest analysis models were developed to estimate the PMI based either uniquely on 16s rRNA gene microbial data derived from nasal swabs or based on both microbial data and measurable environmental parameters such as snow depth and outdoor temperatures, on a total of 393 samples.

**Results:**

Among the six developed models, the best performing one was the complex model based on both internal and external swabs. It achieved a Mean Absolute Error (MAE) of 1.36 weeks and an R2 value of 0.91. On the other hand, the worst performing model was the minimal one that relied solely on external swabs. It had an MAE of 2.89 weeks and an R2 of 0.73. Furthermore, among the six developed models, the commonly identified predictors across at least five out of six models included the following genera: *Psychrobacter* (ASV1925 and ASV1929), *Carnobacterium* (ASV2872) and *Pseudomonas* (ASV1863).

**Discussion:**

The outcome of this research provides the first microbial model able to predict PMI with an accuracy of 9.52 days over a six-month period of extreme winter conditions.

## Introduction

The postmortem interval (PMI) is the time elapsed between death and the body discovery, and its estimation is extremely important in the court of law in cases of homicide and suspicious deaths (Janaway et al., 2009). The decomposition process of an animal or a human body can be divided into five stages of decay based on physical and chemical changes (Smith, 1986). The early postmortem physical changes commonly documented are temperature changes (algor mortis), muscular contraction/relaxation (rigor mortis), and the pooling of blood by gravity after blood circulation stops (livor mortis) (Janaway et al., 2009). Following the early postmortem changes, the decomposition of a body progresses through several stages that lead to complete skeletonization. The first stage is the “fresh,” characterized by the autolytic self-digestion of cells and the start of the putrefactive processes caused by the bacterial breakdown of tissues. Following, “bloating” (or putrefaction) is the stage when the accumulation of gasses produced by bacteria causes the body to bloat and to discolorate. Then, “active decay” involves the piercing of the body, which releases the accumulated gasses, and the breakdown of tissues, organs, and bodily fluids. It is characterized by a strong and distinctive odor and the liquefaction of tissues. During active decay, most of the body mass is lost due to the action of larvae feeding on the remains (Smith, 1986). Subsequently, “advanced decay” is characterized by the presence of leathery skin, few bones, hair, and other resistant materials. Finally, “skeletonized” (or dry remains) is the stage when only skeletal remains and any non-degradable materials such as dental fillings or artificial joints are available (Teo et al., 2014). The tissues progress through these stages faster during higher temperatures, while the lower temperatures can cause a delay or even can halt decomposition, making the PMI estimation challenging (Carter et al., 2015). Consequently, the classification of the decomposition stages may not be relevant in environments with extremely low temperatures.

Among the key players driving decomposition, intrinsic microorganisms (e.g., those that naturally inhabit various tissues and organs of the human body) and extrinsic microbial populations (e.g., foreign invaders from the environment) were shown to have an essential role as decomposers, due to their capability to start the decay process and to make it progress further (Hyde et al., 2013, 2015; Carter et al., 2015; Metcalf et al., 2016). Moreover, the microbial succession on cadaveric remains can be successfully linked with PMI, therefore offering a molecular tool also known as “microbial clock” able to estimate the time elapsed since death with great accuracy (Metcalf et al., 2013, 2017; Javan et al., 2016; Roy et al., 2021). Researchers have made attempts to study more systematically the decomposition process and the associated microbial successions in various circumstances (e.g., different host species, environments, burial conditions, anatomical locations for samplings, etc.) to finely tune the microbial clock and to evaluate its applicability to forensic scenarios / death investigations where PMI is unknown (Pechal et al., 2014, 2018; Guo et al., 2016; Burcham et al., 2019; Iancu et al., 2023).

While several works have focused on the comparison between animal and human remains and have successfully demonstrated the possibility of using animal analogs to study the postmortem microbial succession, there is very scarce information availability on decomposition in extreme negative temperatures (Komar, 1998; Cockle and Bell, 2017; Alfsdotter and Petaros, 2021).

Studies (Carter et al., 2008; Bucheli and Lynne, 2016; Guo et al., 2016; Metcalf et al., 2016; Tozzo et al., 2020; Roy et al., 2021) so far have primarily focused on decomposition during elevated temperatures, using either outdoor locations or controlled laboratory conditions. It is well known that the decay rate is influenced by many factors, including the cause of death, environment, season, vertebrate and invertebrate scavengers, humidity, oxygen content, precipitation, and temperature (Janaway et al., 2009). Among all factors, environmental temperature plays a crucial role for insect activity, microbial composition, and rate of the decomposition process. Thus, during low temperatures, insects are no longer present on the body, while extreme low temperatures together with snow depth can limit the activity and access of vertebrate scavengers (Catts and Goff, 1992). Microorganisms are consistently present in wide ranges of temperatures during decomposition, as such, these could be the only witnesses for the PMI estimation (Iancu et al., 2015; Bucheli and Lynne, 2016; Metcalf et al., 2017). Certain bacteria species, like the ones belonging to *Psychrobacter* genus are better adapted to cold environments, being identified during decomposition (Iancu et al., 2015).

The main objective of the current research was to answer the following critical question: can forensic microbiology assist in predicting the PMI for bodies disposed of in an open field, exposed to extreme environmental factors (temperature as low as −39°C, and snow depth as high as 130 cm)?

Consequently, the current research aimed to fill in this severe knowledge gap on decomposition in extreme environments, by providing for the first-time comprehensive data related to the decomposition process of pig carcasses along 6 months of extreme winter temperatures in a North Dakota outdoor location. This work will analyze the evolution of the stages of decomposition in association with the climatic conditions and will evaluate the necrobiome structural patterns during the carcasses’ breakdown. Additionally, this research will evaluate two different sampling areas for the successful development of a microbial clock applicable in such extreme environments and will provide the first microbial model able to predict PMI with an accuracy of 9.52 days in severe negative temperature environments.

## Materials and methods

### Experimental design

Three pig carcasses (*Sus domesticus* Erxleben, 1777) (approx. 50 kg each) were purchased from a local pig farm and used as human analogs for the decomposition and microbiome investigation. The Institutional Animal Care and Use Committee (IACUC) protocol was not required, as the pigs were euthanized at the farm by captive blitz bolt. Further, the carcasses were transported to the research site in less than an hour and placed on the Mekinock Field Station research land (47°57′11.5″N 97°25′42.4″W), University of North Dakota, Grand Forks, North Dakota, at 20 m from one another, facing south. All three carcasses were protected from vertebrate scavengers by cages (120 × 90 × 180 cm).

University of North Dakota Field Station Committee approved the use of a designated plot for decomposition studies, while the research project received the approval from the Institutional Biosafety Committee (IBC) University of North Dakota (IBC-202111-009).

Daily temperatures (minimum and maximum), relative humidity, and wind speed were recorded from the nearest weather station from the research site (Grand Forks Air Force Base Weather Station, ND), at 3 km, respectively. The temperature under the snow was recorded during each sampling time.

### Sample collection

Sample collection was performed weekly, for 23 weeks, starting in mid-November 2021. Tissue samples were collected in triplicate via sterile cotton swabs from two regions of the head area, as follows: exterior region of the nostrils (circular swabbing for 30 s); interior region of both nostrils (circular swabbing 15 s/nostril). The sampling areas were selected based on accessibility under dense snow cover. To avoid disturbing the entire carcass, only the front part of the cage was excavated during sampling, and it was carefully covered again afterward. A total number of 402 samples were preserved in sterile tubes (without any buffer) at −20°C until further analysis. Pig number 2 (P2) was not sampled during week 11 due to the ice thickness and extreme field conditions; and pig number 1 (P1) was not sampled during week 21 because the head was submerged, as the field was partially flooded from the melting snow.

The carcasses were most of the time under a thick layer of snow, monitored and recorded weekly. During each sampling, the head area was uncovered only for the duration of samples collection, and covered again with snow, to not influence the local decomposition environment.

### Genomic DNA isolation

A modified Qiagen Blood and Tissue protocol was used for genomic DNA isolation. The protocol used double the quantity of the lysis buffers, buffer ATL 360 μL/sample, and buffer AL 400 μL/sample. This modification was chosen because the swabs were not preserved in any buffer, and there was an increased risk of them absorbing the initial lysis buffers and drying during the incubation step. NanoDrop One spectrophotometer (Thermo Scientific, United States) was used to assess DNA concentration and purity, using the ratio of absorbance at 260 and 280 nm. To avoid any contamination and cross contamination of the samples during collection, sterile gloves, cotton swabs, and collection tubes were used, while all laboratory work was performed under aseptic conditions via a purifier filtered PCR (Polymerase Chain Reaction) enclosure (Labconco, United States). The isolated DNA samples were stored at −20°C until submission for Illumina MiSeq sequencing.

### 16S rRNA gene sequencing and processing

Samples were sequenced via Illumina MiSeq PE300 sequencing platform, using the primer pair 341F/805R for the PCR amplification of the 16S rRNA gene fragments (V3–V4 variable region) [100 K reads per sample/amplicon], at the McGill Genome Centre, Canada.

Paired-end reads from each sample were sequenced with forward and reverse reads in separate files and processed by means of the microbiome bioinformatics platform QIIME2 (Quantitative Insights Into Microbial Ecology 2), v.0.99.6 (Bolyen et al., 2019). Nine samples that failed the sequencing were excluded from further processing, resulting in 393 samples being used for data analysis. Denoising and quality control, including removal of chimeras, were achieved by means of the DADA2 plugin v. 1.26.0 (Callahan et al., 2016) and to avoid low quality sequences reads were truncated to (280 bp for forward, 220 bp for reverse reads). The classifier adopted for the taxonomic assignment was Silva v.138 (99% OTUs full-length sequences) (Quast et al., 2012). Statistical analyses were performed within the computing environment R (R Core Team, 2021). All the taxon abundances were calculated and graphically plotted with the aid of the “phyloseq” v.1.42.0 package (McMurdie and Holmes, 2013). Alpha diversity was employed to evaluate differences within different individuals, locations, and snow coverage between the samples. Significance was tested via global Analysis of Variance (ANOVA) and pairwise *t*-test with α < 0.05. Beta diversity for differences between the same groups was investigated by means of Principal Coordinate Analysis (PCoA).

### Modeling

PMI was modeled by means of random forest (RF) implemented in the ‘ranger’ package v0.15.1 (Wright and Ziegler, 2017). The sample was divided into 70% development (train) and 30% validation (test) set maintaining balanced class distributions according to the entire PMI range (23 weeks). Three models were trained on different sample subsets: total sample (train *N* = 271, test *N* = 114), swabs obtained from internal nasal cavity (train *N* = 139, test *N* = 58), and swabs obtained from external nasal cavity (train *N* = 133, test *N* = 55). Model tuning was performed based on *i*th hyperparameter combination based on the following grid:hyper_grid <− expand.grid(.
num.trees = floor(n_features / c(10, 20, 30, 40, 50)),
mtry = floor(n_features * c(0.05, 0.15, 0.25, 0.333, 0.4)),
min.node.size = c(1, 3, 5, 10),
replace = c(TRUE, FALSE),
sample.fraction = c(0.5, 0.6, 0.7, 0.8, 1),
rmse = NA.
)


The models were then used to estimate PMI on the validation set. Root mean square error (RMSE), absolute error (MAE), and correlation coefficient (R^2^) were used to compare the different model performance on both development (out-of-bag) and validation set results. Variable of importance (VIP) was considered to identify ASV with estimation power using importance scores based on permutation. Results were visualized in “ggplot2” v3.4.4 (Wickham, 2016). The same modeling approach was reproduced adding timepoint temperature and snow coverage as independent variables to evaluate the difference in performance when environmental conditions are known.

## Results

### Environmental conditions and decomposition

The decomposition site is situated on the Mekinock Field Station research land, University of North Dakota, Grand Forks, ND, 2 km North of US 2 highway (Supplementary Figure S1). The site is situated between agricultural fields, with corn, soybeans, and wheat crops, and it is characterized primarily by tallgrass prairie. For six winter months the field has been covered with snow varying in depths, up to 130 cm.

Grand Forks environmental conditions are characterized by bitter cold temperatures, and high winds, being cataloged as the second coldest location in the US, after Fairbanks, Alaska (https://www.weather.gov/wrh/Climate?wfo=fgf, n.d.). Another important environmental characteristic is represented by the high difference between the minimum and maximum daily temperature.

During the current experiment, three pig carcasses were placed in the field mid-November, being covered with snow shortly after. The last half of November recorded a maximum temperature of 5°C, while most of the temperatures recorded in December were negative, dropping to −33°C. January recorded freezing temperatures, with a minimum record of −38°C, and February followed with a similar pattern. March continued with freezing temperatures as low as −26°C, while the second half of the month registered warmer temperatures. April recorded a minimum of −16°C and a maximum of 13°C (Supplementary Figure S2).

The relative humidity was constant within the same month and between months (Supplementary Figure S3). The most frequent precipitation rates were recorded for January and February. Snow was the main form of winter precipitation, sometimes accompanied by freezing rain, ice, and sleet. During the experimental time frame the snow depth recorded 130 cm. High winds up to 50 km/h were recorded in November and February, with a maximum wind speed record of 80 km/h (Supplementary Figure S4).

The decomposition process during the winter months has been characterized by a freezing state. At the end of November, the outlining of the superficial blood vessels could be observed in the abdominal area of pig one, with no visible changes for all the winter weeks that followed, as the carcasses were covered with snow. At the end of March, the snow melted, followed by another snowstorm in April. While the current experiment focused on investigating the months with extreme winter temperatures, the decomposition process was monitored until skeletal remains stage, recorded mid-June.

### Microbial taxonomic diversity and abundances

The 393 samples gave a total of 18,153,409 raw sequences, with 46191.9 mean reads per sample. After the denoising step, 10,377,810 high-quality sequences remained. Originally 4,285 Amplicon Sequence Variants (ASV) were identified. Prior to performing formal analyses and creating the figures, pre-processing steps were applied to the ASV counts, including pruning to remove samples with all empty values (total sum of intensities was zero) that results in the additional removal of eight samples. The abundances were standardized to the median sequencing depth according to McMurdie and Holmes (2014); ASVs recognized as mitochondrial, or chloroplast sequences were also excluded. This filtration step resulted in the final identification of 4,173 ASV.

**Figure 1:**
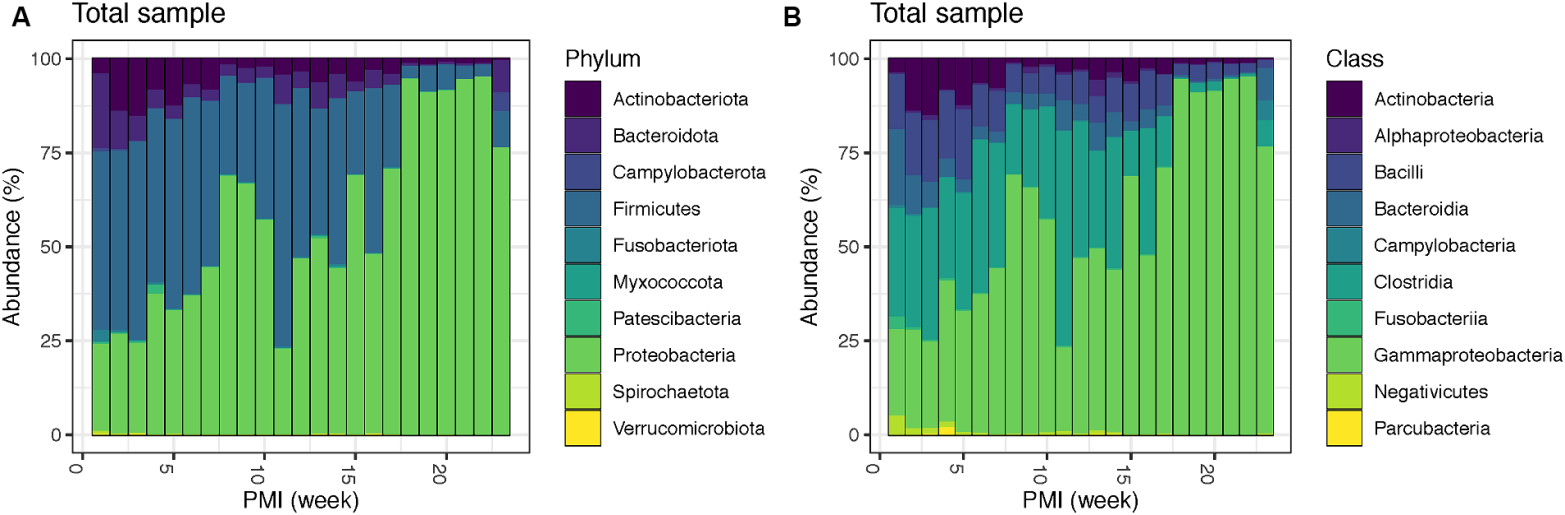
Bacterial community relative abundances for the top 10 phyla **(A)** and classes **(B)** for the total swabs (internal and external) for up to 23 weeks PMI.

Bacterial relative abundances at phylum and class level throughout the decomposition in the total sample (both internal and external swabs) show that Firmicutes, particularly the classes Clostridia and Bacilli, dominate as the most prevalent phylum in the initial weeks (1–7), accounting for an average of 48.8%, followed by Proteobacteria (mainly Gammaproteobacteria) (average 32.3%), Actinobacteriota (mainly Actinobacteria) (average 9.9%) and Bacteroidota, with class Bacteroidia (average 7.3%). Interestingly, after one PMI week, Bacteroidota levels (third most abundant phylum in week one) drop from 20.1 to 10.1%, and Actinobacteriota rise from 3.8 to 13.9% making them the third most abundant phylum in weeks 2–7. At week 8 there is a significant increase in Proteobacteria, which becomes 68.9% of the total population. Proteobacteria remains the dominant phylum until week 10, with a notable decrease in week 11 (22.9%). From weeks 12 to 16, Proteobacteria (average 52.0%) and Firmicutes (average 37.7%) have similar relative abundances, whereas from week 17 onwards Proteobacteria becomes the most abundant phylum until the end of the experiment (average 87.8%) (Figure 1A). Remarkably, there is also an increase in Campylobacterota with the class Campylobacteria (4.9%) and Bacteroidota (8.8%) in week 23 (Figure 1B). Details for the internal and external samples at phylum and class level can be found in the Supplementary Figure S5.

### Microbial alpha and beta diversity analyses

**Figure 2:**
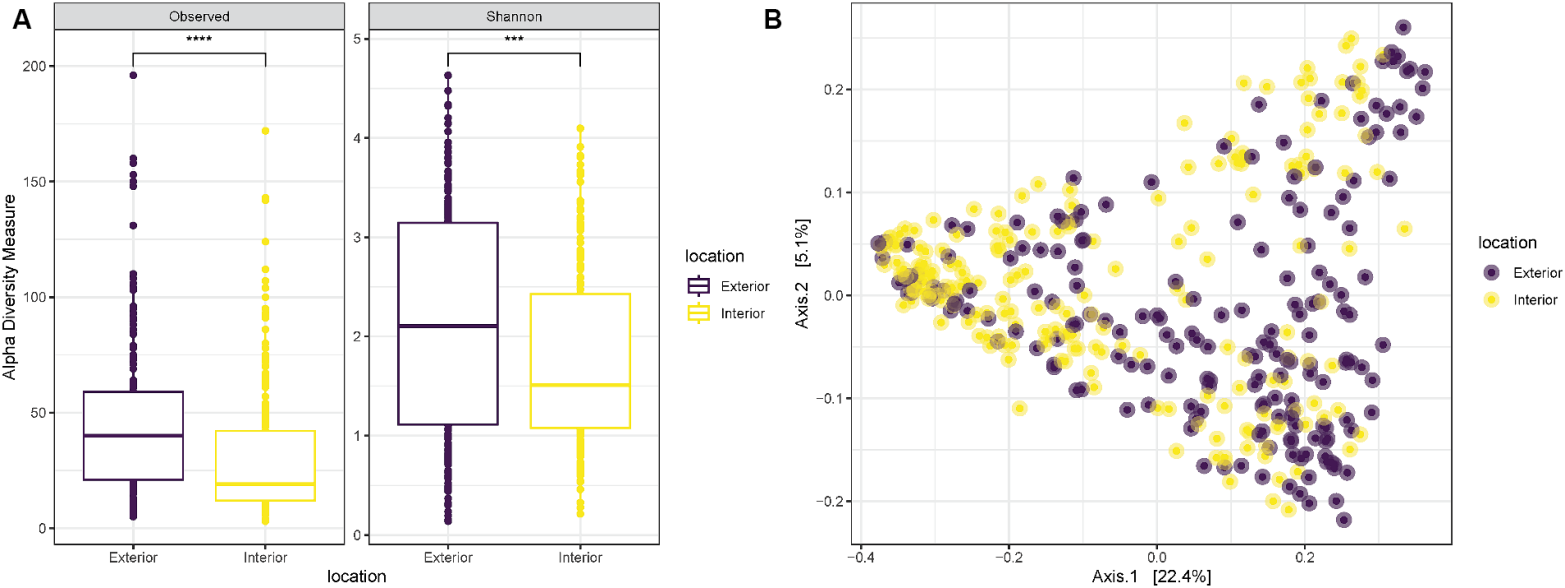
**(A)** Observed richness and Shannon diversity for samples collected either externally (purple) or internally (yellow) from the nostrils of the three pigs. *p*-value significance: * <0.05, ** <0.01, *** <0.001, **** <0.0001. **(B)** PCoA (Principal Coordinates Analysis) on unweighted-UniFrac distance for samples collected externally (purple) or internally (yellow) from the nostrils of the three pigs.

Alpha diversity measurements to compare the two anatomical locations used for the samplings revealed a significantly higher richness (“Observed”) and diversity (“Shannon”) for samples collected on the exterior part of the nose in comparison with those collected internally (*T*-test *p*-value < 0.0001 and 0.00058, respectively) (Figure 2A). Despite the increased diversity of the exterior samples, when plotted on a PCoA all samples were equally spread and did not create specific clusters associated with the anatomical location of the sampling (Figure 2B).

**Figure 3:**
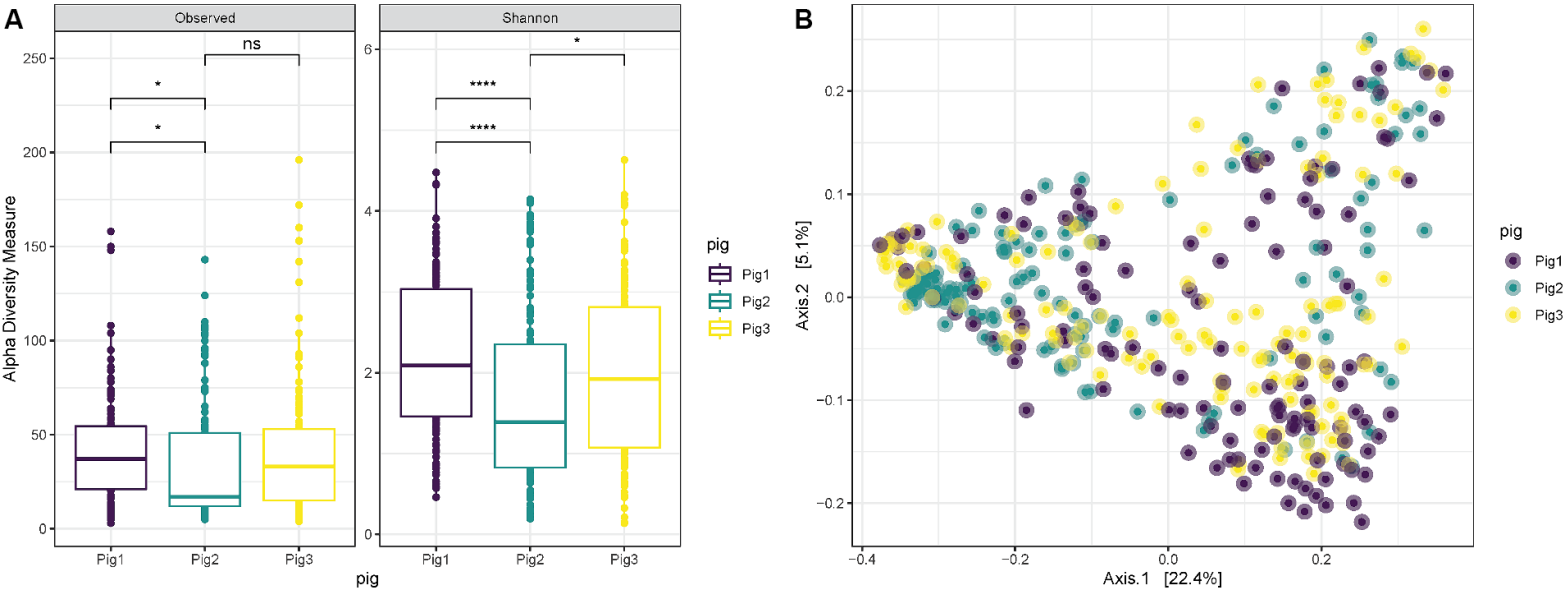
**(A)** Observed richness and Shannon diversity for samples collected both internally and externally from Pig 1 (red), Pig 2 (gray) and Pig 3 (blue). *p*-value significance: * <0.05, ** <0.01, *** <0.001, **** <0.0001. **(B)** PCoA (Principal Coordinates Analysis) on unweighted-UniFrac distance for samples collected from Pig 1 (red), Pig 2 (gray) and Pig 3 (blue).

When comparing the diversity results for the three pigs used in the experiment, it is possible to notice that “Pig 2” has a lower richness (ANOVA *p* = 0.0088) and Shannon diversity (ANOVA *p* = 0.00042) in comparison with “Pig 1” (Figure 3A). However, also in this case the overall distribution of the samples collected from the three animals in the PCoA does not show any clustering associated with the specific animal, and “Pig 2” samples are spread and mixed between “Pig 1” and “Pig 3” samples (Figure 3B).

**Figure 4:**
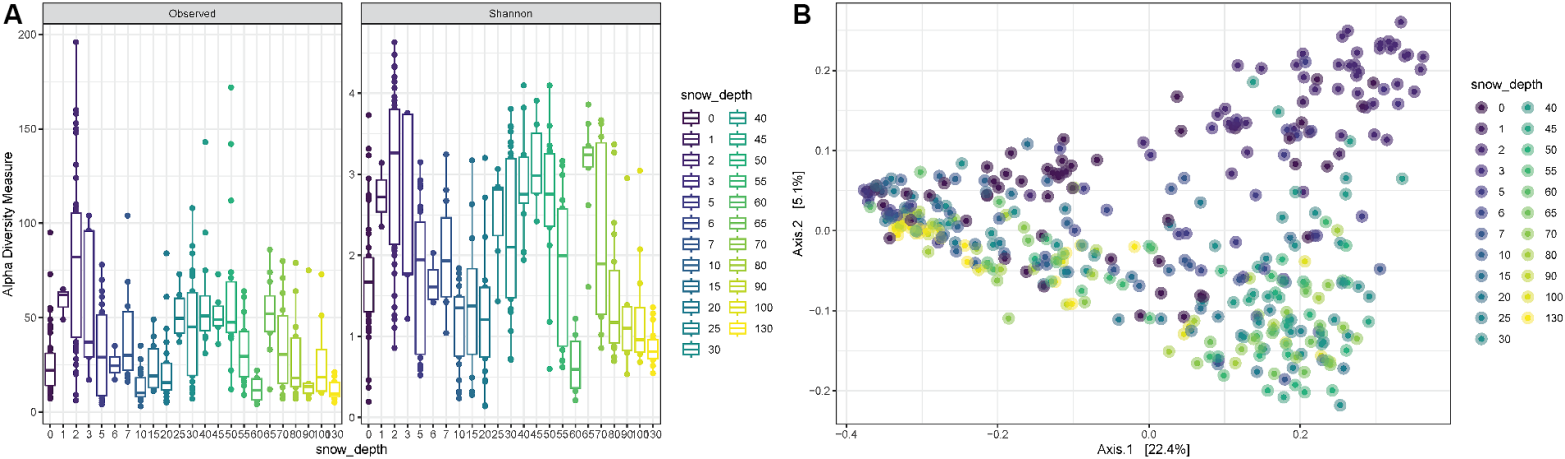
**(A)** Observed richness and Shannon diversity for samples collected under increasing snow depths in cm. **(B)** PCoA (Principal Coordinates Analysis) on unweighted-UniFrac distance for samples collected under different snow depth levels.

Snow depth was also considered when evaluating alpha diversity during all 23 weeks. The observed richness, as well as the Shannon diversity, fluctuates with a tendency of higher mean values for very shallow depths (~5–17 cm) and for medium depths (~63–140 cm) and lower means for small depths (~25–50 cm) and large depths (~177–130 cm) (ANOVA *p*-value < 0.0001) (Figure 4A). The PCoA shows consistent trends according to the snow coverage, suggesting that certain variables are differentially abundant at different depth conditions (Figure 4B).

**Figure 5:**
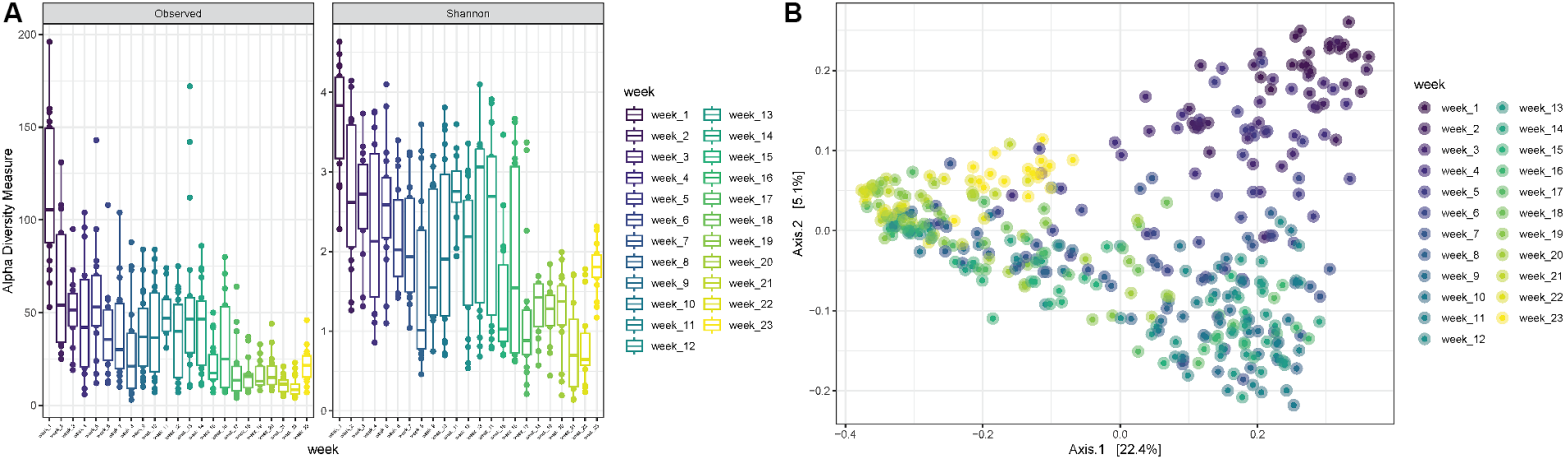
**(A)** Observed richness and Shannon diversity for samples collected for increasing PMIs (reported in weeks). *p*-value significance: * <0.05, ** <0.01, *** <0.001, **** <0.0001. **(B)** PCoA (Principal Coordinates Analysis) on unweighted-UniFrac distance for samples collected at increasing PMIs from 1 to 23 weeks.

Finally, when looking at increasing PMIs, it is possible to observe a decrease in observed richness as well as in Shannon diversity up to 8 weeks, followed by some fluctuations with increased diversities from 9 to 14 weeks, after which both observed richness and Shannon diversity decrease again (ANOVA *p*-value < 0.0001) (Figure 5A). The PCoA shows consistent trends according to the PMI, suggesting that certain variables are differentially abundant at specific time points postmortem (Figure 5B).

### Postmortem microbiome prediction model

**Figure 6:**
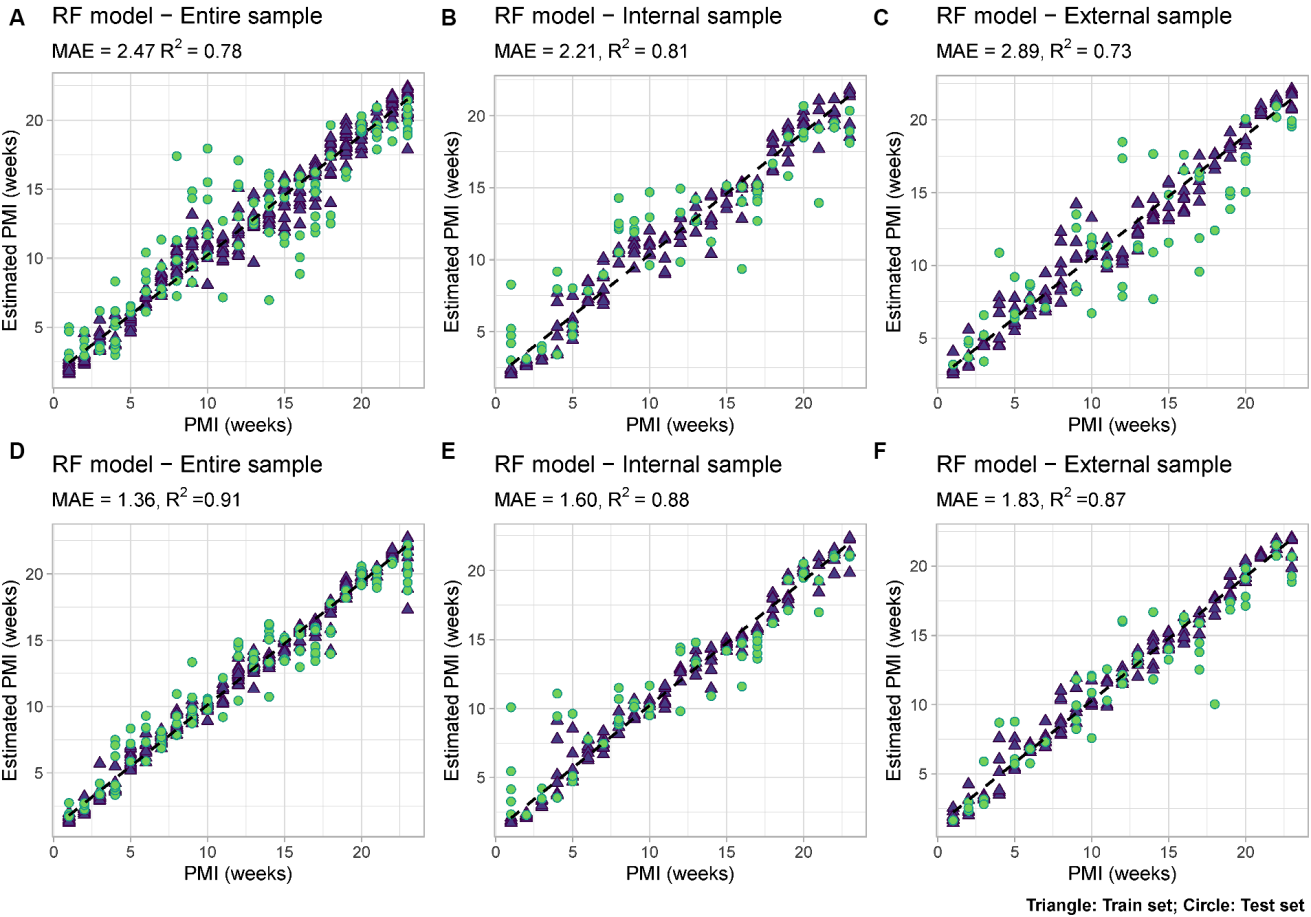
RF models based on: **(A)** Microbial data only (“Minimal RF Model”) using internal and external swabs (“Total”); **(B)** Microbial data only (“Minimal RF Model”) using internal swabs only (“Internal”); **(C)** Microbial data only (“Minimal RF Model”) using external swabs only (“External”); **(D)** Microbial and environmental data (“Complex RF Model”) using internal and external swabs (“Total”); **(E)** Microbial and environmental data (“Complex RF Model”) using internal swabs only (“Internal”); **(F)** Microbial and environmental data (“Complex RF Model”) using external swabs only (“External”). Mean Absolute Error (MAE) and *R*^2^ values are reported for each model. Train set = blue triangle; test set = green circle.

Using Random Forest analysis models were developed to estimate PMI based either uniquely on the bacterial data (“Minimal RF Model”) or based on both microbial data and measurable environmental parameters such as snow depth and external temperature (“Complex RF Model”). For each of them, models were developed either using only the internal nose swabs (“Internal”), the external nose swabs (“External”) or using all the swabs (“Total”) (Figure 6).

**Figure 7:**
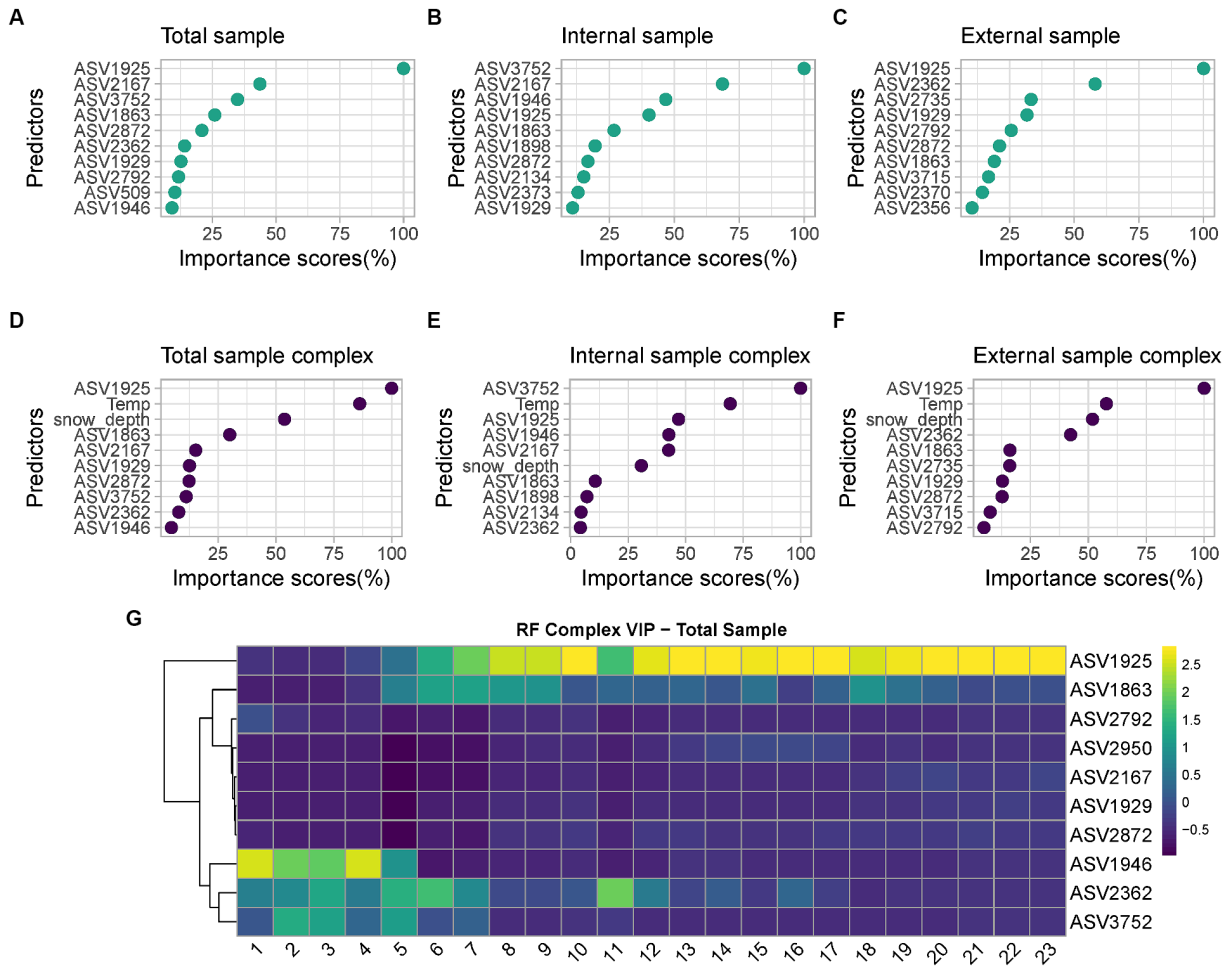
List of predictors with importance scores >1 used by: **(A)** “Minimal RF Model” with “Total” samples; **(B)** “Minimal RF Model” with “Internal” samples; **(C)** “Minimal RF Model” with “External” samples; **(D)** “Complex RF Model” with “Total” samples; **(E)** “Complex RF Model” with “Internal” samples; **(F)** “Complex RF Model” with “External” samples. For the taxonomic assignation of each ASV see Supplementary material Data S1; **(G)** Heatmap of the VIP selected by the “Complex RF Model” with “Total” samples indicating their relative abundances over increasing PMIs.

Among the six developed models, the best performing one is the complex model based on both internal and external swabs (MAE = 1.36 weeks, *R*^2^ = 0.91), whereas the worst performing one is the minimal one based on external swabs only (MAE = 2.89 weeks, *R*^2^ = 0.73). The variable importance plots (VIPs) with a score > 1 showed the consistent presence of specific predictors across all the different models developed (ASV1925, ASV1863), across the majority of the models (ASV2872, ASV1929), and across specific models only (ASV2167, ASV3752, ASV1946 only in “Total” and “Internal” models, ASV2134, ASV2373, ASV1898 only in “Internal” models, ASV2362, ASV2792, ASV2356 only in “Total” and “External” models, ASV2735, ASV2370, ASV2845, ASV3715 only in “External” models, ASV2950, ASV509 only in “Total” models) (Figure 7). For the “Complex” models, temperature and snow depth always represented predictors with a high importance score.

Among all predictors identified, those commonly identified between at least five out six models include the genera *Psychrobacter* (ASV1925 and ASV1929), *Carnobacterium* (ASV2872) and *Pseudomonas* (ASV1863). Other taxa were defined as good predictors only in selected models; examples are the genera *Aeromonas* (ASV2167), *Rothia* (ASV3752), *Moraxella* (ASV1946), *Clostridium_sensu_stricto_5* (ASV2373), *Pseudomonas* (ASV1898) and *Shewanella* (ASV2134), which are considered identifiers only in “Internal” or “Internal” and “Total” models. Similarly, the genera *Clostridium_sensu_stricto_1* (ASV2362 and ASV2356, including the species *Clostridium_butyricum,* ASV2370), *Lactobacillus* (ASV2792), *Streptococcus* (ASV2735), *Terrisporobacter* (ASV3715) and *Turicibacter* (ASV2845) are identifiers only for “External” or “External” and “Total” models. Finally, genus *Sporosarcina* (ASV2950) and *Bacteroides_stercoris* species (ASV509) are identifiers only in “Total” models.

Considering the most significative taxa for PMI estimation based on their importance scores, it is possible to identify different trends (Figure 7G); respectively, taxa whose relative abundance increases over time, and taxa characterized by an initial increase on short PMIs followed by a decrease in abundance over time. *Psychrobacter* sp. increase in abundance consistently starting from 5 weeks PMI and reach and maintain their highest relative abundance starting from 10 weeks PMI. Similarly, *Pseudomonas* sp. tend to increase in abundance between 5 and 9 weeks PMI and are high again at 18 weeks. On the contrary, *Moraxella* sp. are abundant from 1 to 5 weeks, after which their abundance is low. *Clostridium_sensu_stricto_1* sp. abundance is relatively high from 1 to 7 weeks, decreases in weeks 8–10, and increases again in weeks 11–16, after which decreases to low levels. *Rothia* sp. finally increased in abundance from 2 to 5 weeks PMI and constantly decreased until the end of the experiment.

## Discussion

The estimation of the time elapsed since death is a highly exploited topic in the forensic arena, with an increasing body of research being conducted with the ultimate aim of improving the current understanding on cadaveric decomposition. One key research area is the development of models for PMI estimation applicable to various scenarios (e.g., terrestrial, or aquatic environments, exposed or buried bodies) and tuned to consider the effect that intrinsic and extrinsic variables may play on such estimations (Metcalf et al., 2013, 2017; Burcham et al., 2024). Among all the variables known to affect the decomposition rate and consequently the PMI estimation of remains, temperature is probably one of the most studied ones (Mann et al., 1990; Carter et al., 2006, 2008; Burcham et al., 2019). Decomposition is halted by very low temperatures, and macroscopically it can result in the complete interruption of the successional gross changes normally observed with increasing PMIs (Damann and Carter, 2013). As a result, PMI estimation in low or extremely low temperature environments suffers from high errors and may ultimately result in justice miscarriages. Moreover, an environment with extreme temperatures poses certain difficulties when examining a death scene. Consequently, to provide reference data and microbial prediction models for the PMI estimation, taphonomy studies in such environments are more than necessary to have a better understanding of the role the environmental factors play during decomposition.

It is worth noting that decomposition continues even at temperatures below 0°C due to the body salt content, as previously demonstrated (Vass et al., 1992), while the snow can act as an insulate for low temperatures by trapping residual heat (Coulson et al., 1995). At or under 4°C the bacterial activity is slowed down (Micozzi, 1997), resulting in a slower rate of decomposition and an inversed colonization. Namely, bodies exposed to extreme cold will decompose from outside in, and not from the inside (gastrointestinal tract). Our study aimed to characterize the microbial spatial and temporal shifts through decomposition of pig carcasses during North Dakota’s extreme winter. With no nearby mountains or big water bodies, Grand Forks is subject to great temperature variation and to the cold Arctic high-pressure system, with high precipitation rate represented by snow during the winter months.

Studies in environments with extreme low temperatures and high precipitation rates, represented by snow, are scarce (Komar, 1998; Wescott, 2018; Alfsdotter and Petaros, 2021). A recent study performed in Sweden (Alfsdotter and Petaros, 2021) aimed to investigate the taphonomic changes and PMI of human cadavers exposed to outdoor terrestrial and aquatic environments. This study used data from autopsies carried out between 2010 and 2020. The decomposition scoring was performed from photographs and the total body score (TBS) for terrestrial cases was assessed after Megyesi et al. (2005) method. In the current experiment, the TBS assessment was not possible as the carcasses were covered by snow for more than 5 months. The authors (Alfsdotter and Petaros, 2021) used linear regression analysis to evaluate the relationship between decomposition stages and ADD, for cases where the PMI was less than 2 years. The results showed a high correlation between TBS and actual logADD, while the longest PMI without skeletonization was seen in a case exposed during the cold part of the year. Another study using ADD and death investigation cases was performed in Canada (Cockle and Bell, 2017), emphasizing the difficulty of PMI estimation during cold and freezing temperatures (4°C or less). Both Sweden and Canada studies used Megyesi et al. (2005) method for the ADD calculations. However, it is worth noting that all negative temperatures were given a “zero” value, while Cockle and Bell (2017) determined that PMI and ADD were not significant dependent variables for decomposition. The bodies that were exposed outdoors during the entire winter in Canada, needed more temperature and time, to progress through the final decomposition stages. When comparing the current results with these previous studies, there is a gap in adding the snow factor and extreme low temperatures. As Cockle and Bell (2017) emphasized, special consideration should be given to bodies found in freezing or cold environments, when involving the PMI estimation, as these bodies will need more ADD to complete decomposition.

If studies on decomposition in cold climates are sparse, studies involving microbiomes during winter decomposition are even sparser. Microbiome data can add critical information for the PMI estimation, as this estimation becomes less accurate with temperature decreasing. The investigation of the postmortem microbiome can be performed via autopsy cases (Pechal et al., 2018; Iancu et al., 2023), outdoor research facilities (Hyde et al., 2013; Bucheli and Lynne, 2016), and field experiments using pig carcasses as human analogs (Iancu et al., 2015; Matuszewski et al., 2020).

Microbial taxonomic successions observed over time are consistent with the results reported in other works conducted in non-extreme environments (Pechal et al., 2014; Metcalf et al., 2016; Burcham et al., 2024), showing an increase of Gammaproteobacteria with prolonged PMIs and a decrease of Firmicutes (Clostridia class) toward the latest time points analyzed, up to 23 weeks. Firmicutes are normally highly abundant at advanced and skeletonised stages (Pechal et al., 2014; Speruda et al., 2022), however they were more abundant during the earlier PMIs and decreased in abundance consistently to reach notably lower relative abundances after 18 weeks PMI. A decrease in Firmicutes abundances on the head skin from the rupture of mice bodies (active decay) was also observed by Metcalf et al. (2013), in association with an increase of Gammaproteobacteria, similarly to the current study. They also found an increase of Alphaproteobacteria during the latest time points analyzed (from 34 to 48 days PMI); similar findings were found in our study specifically after 13 weeks PMI only for the internal samples and not for those collected on the outside of the nose. Similarly, to this previous study (Metcalf et al., 2013), where Actinobacteria present at time 0 and after 3 days on the skin of belly and head decreased consistently in abundance from 9 days onwards, we noticed a reduction in Actinobacteria over the course of the experiment, particularly from 8 weeks PMI onwards. Overall, these results suggest that the microbial succession identified in extreme cold environments follows similar patterns of the non-extreme environment experiments, but on slower rates due to the environmental conditions altering the decomposition process.

When comparing internal versus external samples in the current experiment, Proteobacteria was prevalent in the internal samples, while Firmicutes dominated the external ones, being also characterized by a higher taxonomic diversity. The presence of Firmicutes on the skin of decomposing remains, together with Bacteroidetes and Fusobacteria, was already reported by Dickson et al. (2011) in submerged remains. *Psychrobacter* and *Pseudomonas* could be considered microbial markers when investigating bodies found in similar environments, while *Clostridium_sensu_stricto_1* could be considered when investigating external skin samples. *Psychrobacter, Pseudomonas* and *Carnobacterium* could be considered candidates for “winter microbial markers” in the same way that *Streptococcus* and *Staphylococcus* were observed to be biomarkers for shorter periods of time, in normal environmental conditions (Pechal et al., 2018; Iancu et al., 2023). *Psychrobacter* has been previously described as a putative winter biomarker for above the ground (Iancu et al., 2015) and grave soil (Carter et al., 2015) decomposition environments, and was found specifically in winter studies on submerged pig heads also by Dickson et al. (2011). In the same study, *Carnobacterium* was also found as an indicator of autumn season, differently from the terrestrial study mentioned above and from the current work.

As previous studies (Johnson et al., 2016; Burcham et al., 2024) mentioned, machine learning microbiome-based models (e.g., random forest regression models) can be used to predict the PMI from different climates and environments. In the current case, among all RF models investigated, the best model was based on both sample types (internal and external), while snow coverage and temperature at the time of the sample collection were among the most important predictors.

This is the first study to characterize microbial diversity and dynamics in the second coldest location in the United States, providing the first microbial model able to predict PMI with an accuracy of 9.52 days in severe negative temperature environments, along 6 months of winter. The limitation of the current study could be represented by the lack of Body Total Score (TBS) data. Since the pig carcasses were covered by snow for almost the entire duration of the experiment, no TBS assessment was possible. In order to provide more information regarding the decomposition process and RF models based on microbial succession, successive and comparative studies across different geographical regions should be performed, to be used as reference data in medicolegal death investigations.

## Data availability statement

The datasets presented in this study can be found in online repositories. The names of the repository/repositories and accession number(s) can be found at: https://www.ncbi.nlm.nih.gov/, BioProject ID PRJNA1052957.

## Ethics statement

Ethical approval was not required for the study involving animals in accordance with the local legislation and institutional requirements because the pigs were euthanized at the farm by captive blitz bolt, hence the Institutional Animal Care and Use Committee (IACUC) protocol was not required.

## Author contributions

LI: Conceptualization, Formal analysis, Funding acquisition, Investigation, Methodology, Project administration, Resources, Supervision, Validation, Visualization, Writing – original draft, Writing – review & editing. AB: Data curation, Formal analysis, Investigation, Methodology, Software, Visualization, Writing – original draft. NP: Data curation, Methodology, Software, Visualization, Writing – original draft, Writing – review & editing.
