# Supplementary material for: Decomposition in an extreme cold environment and associated microbiome—prediction model implications for the postmortem interval estimation
Source: Front Microbiol. 2024 May 13;15:1392716. doi: 10.3389/fmicb.2024.1392716 (PMC11128606; doi:10.3389/fmicb.2024.1392716)
Supplement: Supplementary file 1 [file Data_Sheet_1.pdf]

Supplementary Material

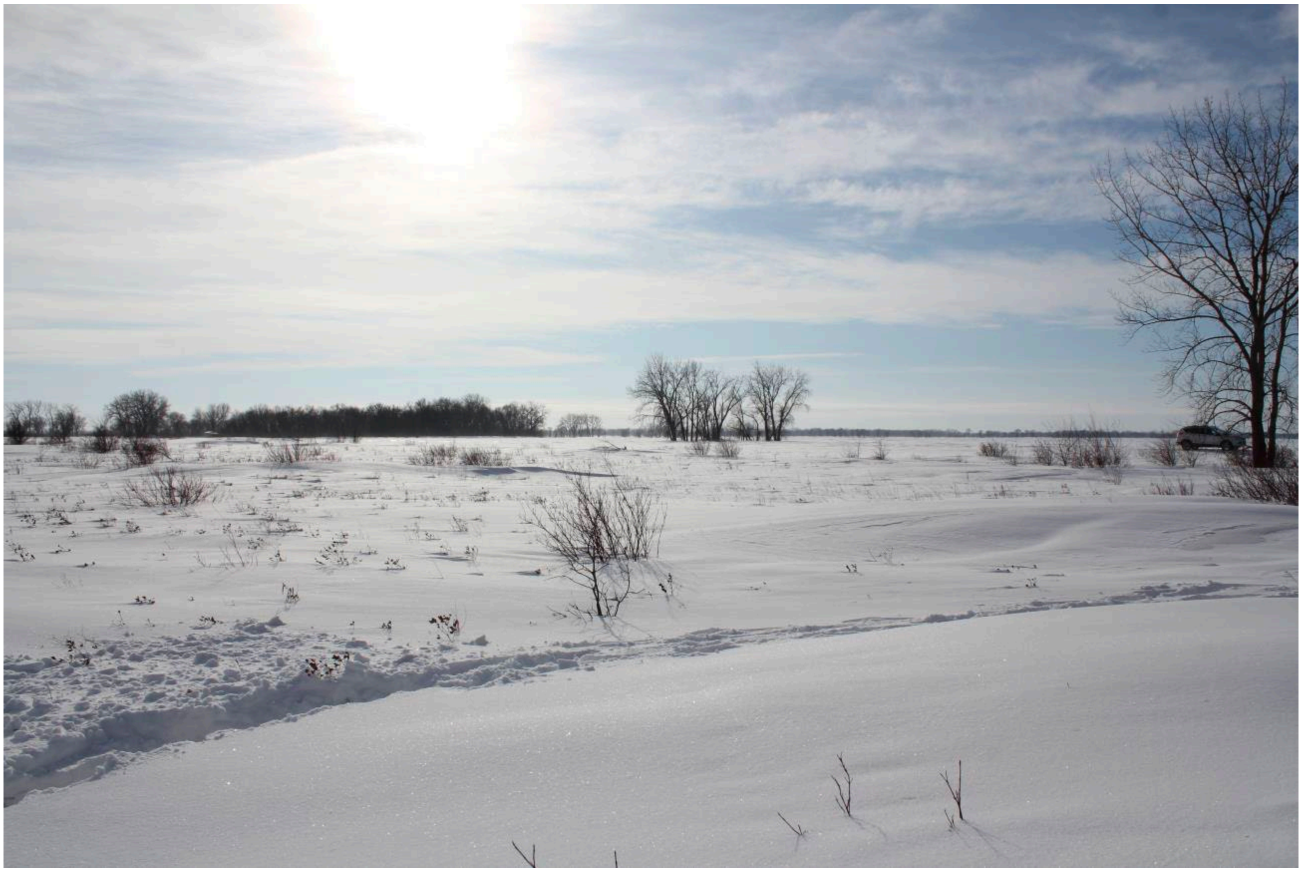

Figure S1.
Decomposition site - Mekinock Field Station, University of North Dakota, Grand Forks, ND.

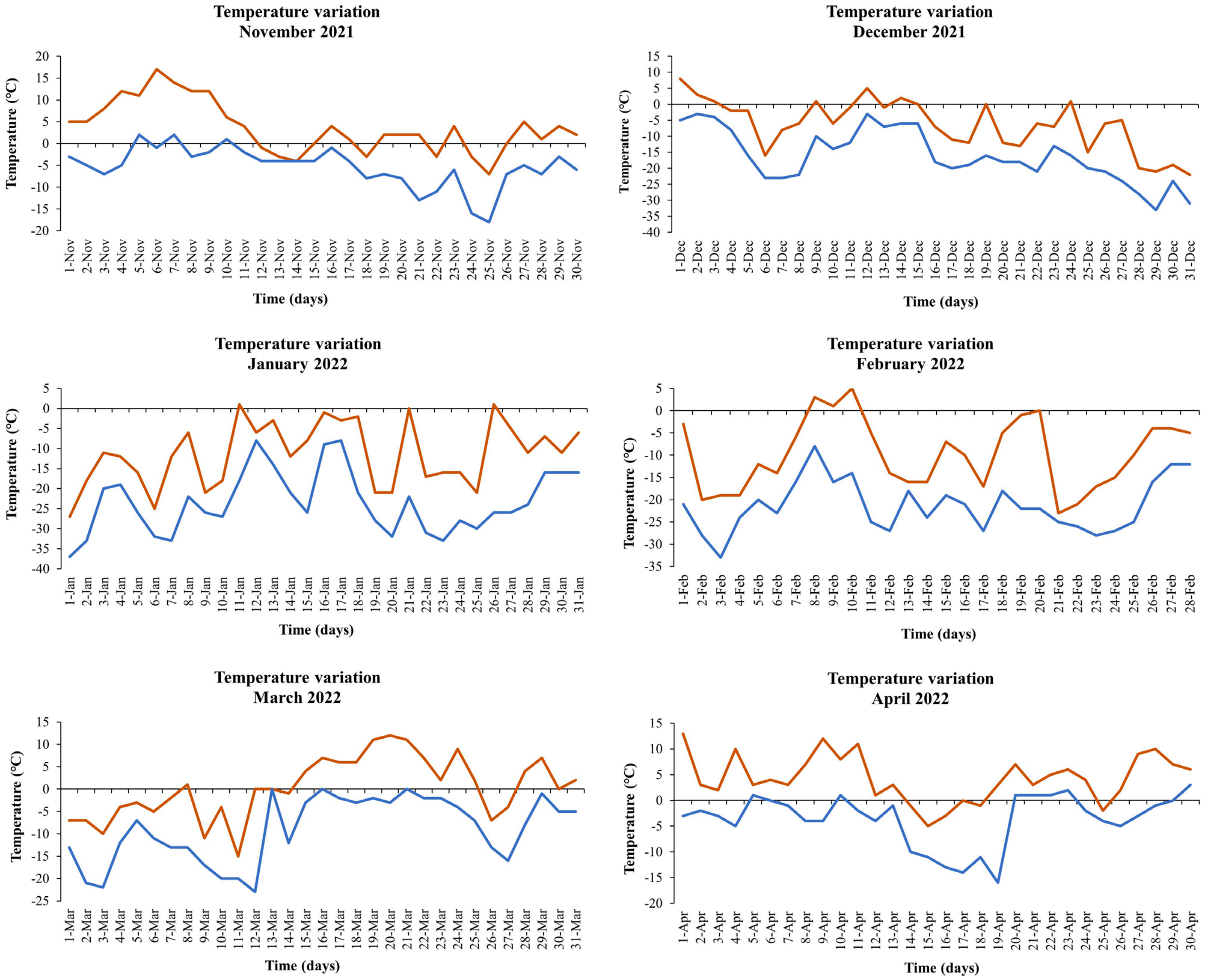

Figure S2.

Temperature variation during the winter experimental timeframe. Red – maximum temperatures; Blue – minimum temperatures.

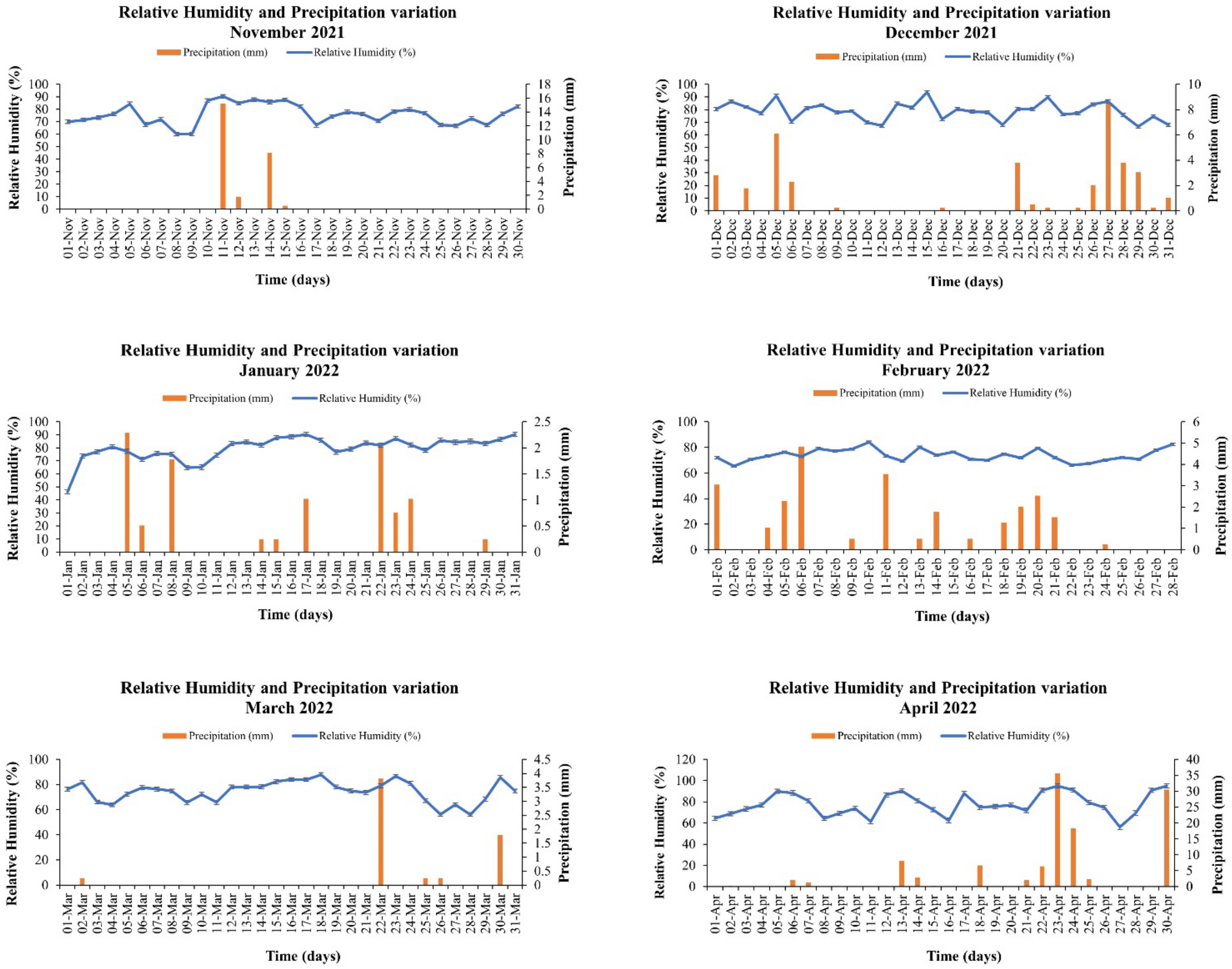

Figure S3.

Relative humidity and precipitation variation during the winter experimental timeframe.

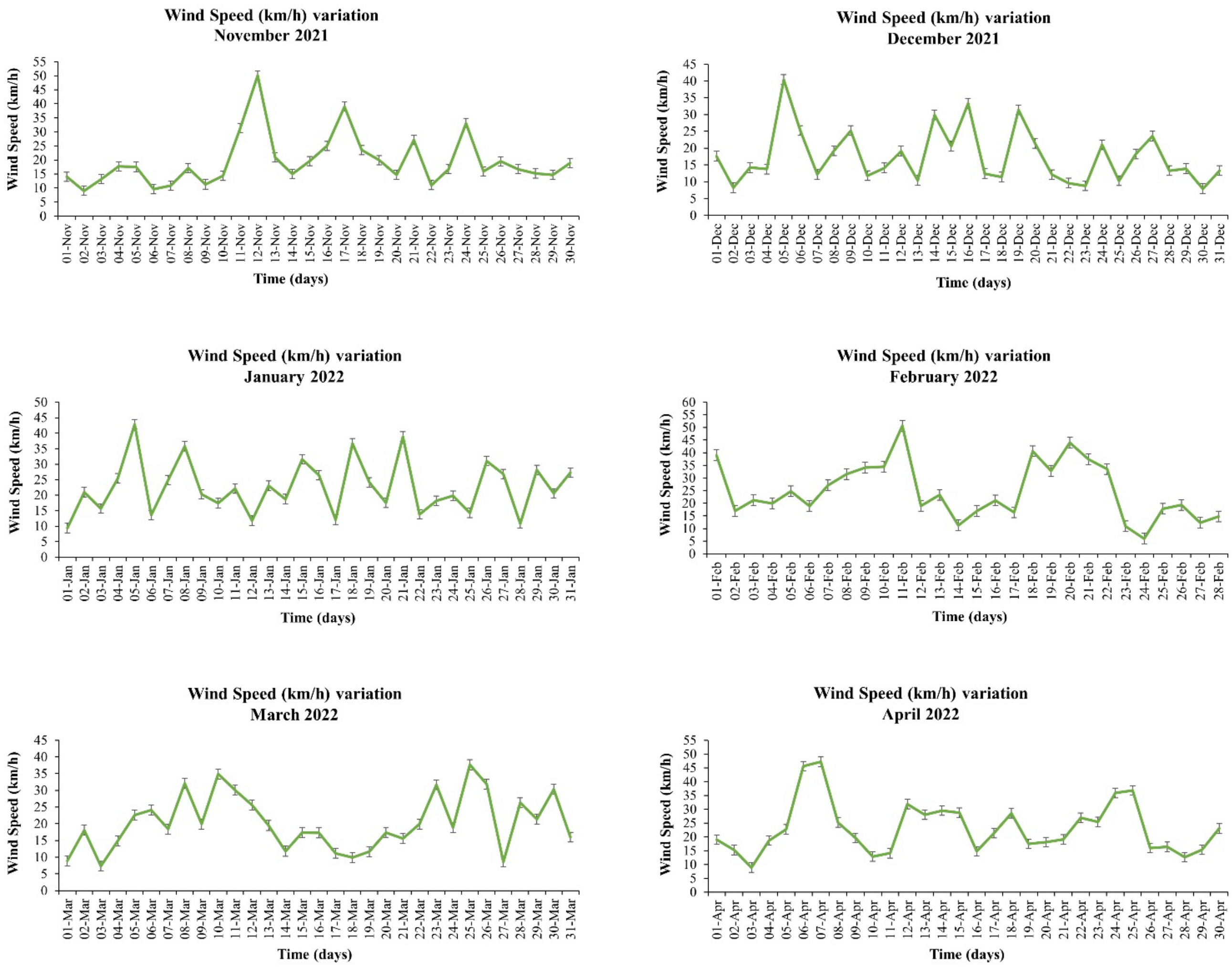

Figure S4.

Wind speed variation during the winter experimental timeframe.

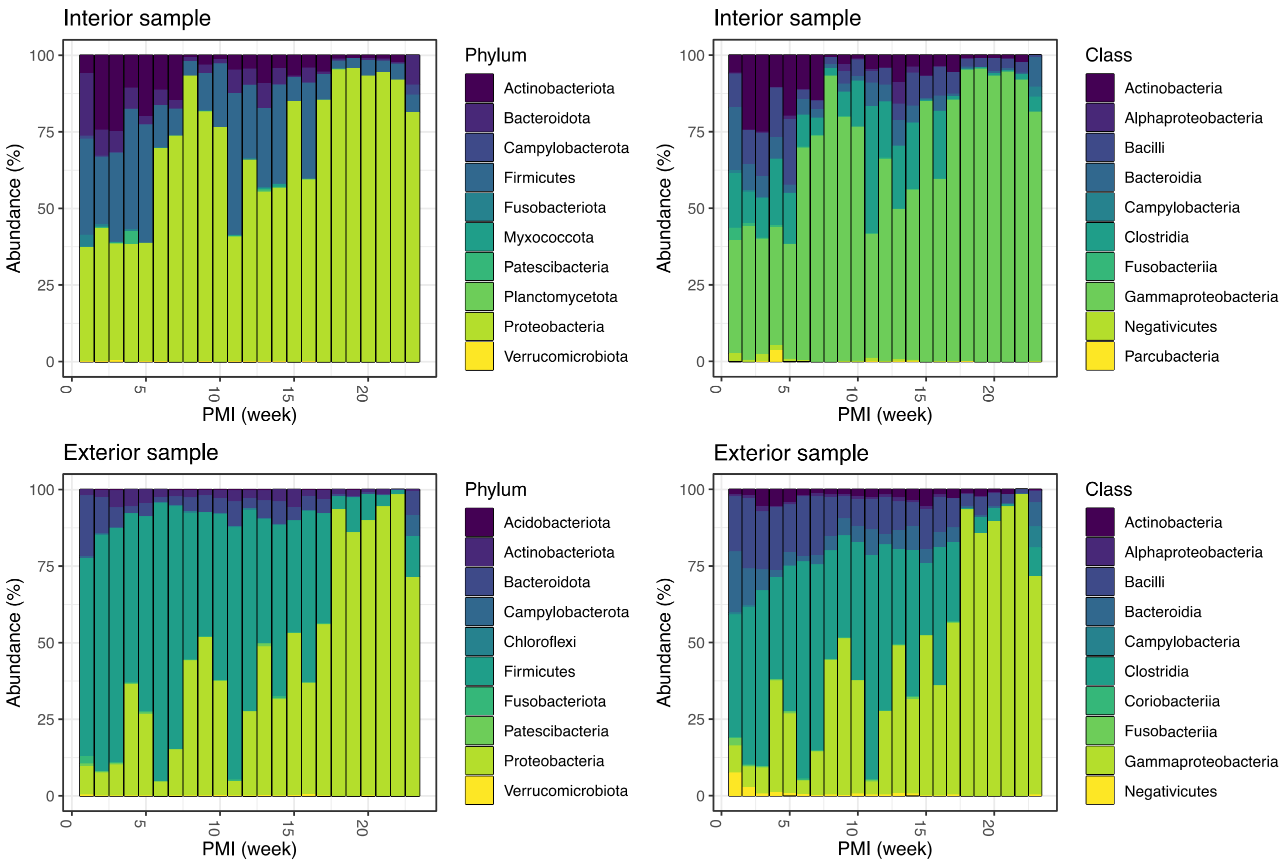

Figure S5.

Bacterial community relative abundances for the top 10 phyla (left) and classes (right) for the internal (top) and external (bottom) swabs for up to 23 weeks PMI.
